# Bidirectional Associations Between Covid-19 Infection And Mental Disorders

**DOI:** 10.1192/j.eurpsy.2022.1264

**Published:** 2022-09-01

**Authors:** M. Turki, S. Blanji, A. Daoud, S. Ellouze, M. Abdellatif, N. Gargouri, N. Halouani, J. Aloulou

**Affiliations:** Hedi Chaker University Hospital, Psychiatry “b” Department, Sfax, Tunisia

**Keywords:** COVID19, psychiatry, Mental Disorders

## Abstract

**Introduction:**

The COVID-19 pandemic brought unbearable psychological pressure to people worldwide, because of serious threats to one’s physical health and life. From early stages of this pandemic, concerns have been raised about its effect on mental health. However, we still know little whether pre-existing psychiatric disorder (PD) affects the susceptibility and evolution of this infection.

**Objectives:**

We aimed to assess the interactions between COVID-19 infection and PD.

**Methods:**

We conducted a litterature review through pubmed database, using the keywords :«COVID 19», «psychiatry», «mental disorders», « schizophrenia », « anxiety », « depression », «insomnia».

**Results:**

On one hand, prior surveys suggested that the infection is associated with increased incidence of a first psychiatric symptom. Mental health disturbances mostly include anxiety, depression, sleep disturbances, cognitive impairment and post-traumatic stress disorder. On the other hand, recent studies showed that patients with pre-existing mental disorders were associated with high susceptibility to be infected, increased risk of intensive care unit admission and a high mortality. The susceptibility to contracting COVID-19 was associated with pre-existing mood disorders, anxiety, and attention-deficit hyperactivity disorder. Infection severity was associated with pre-existing or subsequent mood disorders and sleep disturbances; or a pre-extisting schizophrenia. Mortality is increased in patients diagnosed with schizophrenia.

**Conclusions:**

The complicated interactions between COVID-19 infection and PD have several implications. Enhanced psychiatric follow-up should be considered for survivors of COVID-19. Besides, early detection and intervention for PD are needed to control morbidity and mortality induced by the COVID-19 infection.

**Disclosure:**

No significant relationships.

